# A Review on the Use of the Educational Value Unit (EVU) among Teaching Hospitals

**DOI:** 10.3390/healthcare11010136

**Published:** 2023-01-01

**Authors:** Alina Husain, Darren A. Chen, Gary J. Lelli

**Affiliations:** 1 MD Program, Weill Cornell Medicine, New York, NY 10021, USA; 2 Department of Ophthalmology, Massachusetts Eye and Ear, Boston, MA 02114, USA; 3 Department of Ophthalmology, Weill Cornell Medicine, New York Presbyterian Hospital, New York, NY 10021, USA

**Keywords:** educational value unit(s), academic relative value unit(s), educational productivity

## Abstract

(1) Background: In recent years, medical institutions across the U.S. have implemented a points system based on the Educational Value Unit (EVU) to assess and reward faculty for their educational efforts. The purpose of this narrative review is to summarize the current literature on EVU systems and to evaluate their utility in the U.S. healthcare system. (2) Methods: We searched the Ovid MEDLINE, Embase, Web of Science, and PubMed databases to identify literature describing the inception of EVU systems and current systems implemented by U.S. academic medical centers and medical schools. In total, a combined 48 studies and abstracts pertaining to EVU systems were reviewed, and a combined 26 published studies and abstracts from 1999 to 2022 pertaining to EVU systems were included. (3) Results: To our knowledge, at least 40 U.S. academic medical centers have used an educational metrics system, of which 21 institutions have published studies describing EVU systems in one or more of their medical departments. The outcomes associated with these self-described EVU systems are the focus of this study. EVU systems increase the number of faculty who meet baseline educational requirements, promote educational productivity, redistribute educational burden and funding among faculty members, and shift physician priorities towards education. The monetary reward associated with EVU systems is unlikely to be a significant factor contributing to these changes; instead, intrinsic motivation and a sense of academic responsibility play a larger role. (4) Conclusions: EVU systems are an effective way to evaluate and reward individual and departmental educational efforts in U.S. academic medical centers and medical schools. The adoption of EVUs will likely become more commonplace as U.S. academic medical centers and medical schools place additional emphasis on medical education.

## 1. Introduction

At the core of a successful medical institution is its faculty, who are responsible for providing high quality patient care, educating future medical professionals, and advancing an institution’s academic mission through various educational pursuits. In the process of balancing both clinical and academic obligations, academic physicians face a unique set of challenges. Current payment models compensate physicians according to the number of work relative value units (wRVUs) assigned to various medical procedures [1], which may or may not include research or teaching efforts. Additionally, reimbursement models for physicians have increasingly reduced payment rates, resulting in a need for greater clinical productivity among physicians. Academic physicians must also constantly adjust to evolving medical education pedagogy and compete against their colleagues for limited research funding, which adds additional burden to their high workloads. These factors, among others, contribute to increased burnout among academic physicians [2] and has led to faculty proposing compensation and recognition for non-clinical work as one solution to burnout [3]. Furthermore, a survey of faculty in U.S. academic health centers has shown that faculty vitality, defined by the vibrancy, engagement, and motivation in work pursuits [2], is highest in young faculty and lowest in midcareer faculty members, in whom academic health centers have invested significant training and development. Faculty vitality [4] and satisfaction [5] are strongly predicted by the alignment of individual and institutional values, highlighting the need for institutions to recognize a faculty member’s efforts towards achieving their institution’s educational mission. 

To address a lack of supplemental compensation or recognition for physician teaching efforts and to better align institutional and individual values towards education, departments across various U.S. medical schools and teaching hospitals have implemented a metrics system based on the educational value unit (EVU). EVU systems assess a faculty member’s performance using the sum of the activity or time weighted units for educational activities performed in the academic year [6]. These sums may be used to reallocate funds within [7] or between departments [8] to better support an institution’s educational mission, to determine a year-end financial bonus [9,10], or to reconsider an individual’s faculty status [11], among other things. To our knowledge, at least 40 U.S. academic medical centers have used an educational metrics system [12], and a total of 21 U.S. medical schools and teaching hospitals have published reports describing the EVU system implemented in one or more of their medical departments. Our summary of the literature provides a primer on EVU systems, describes current EVU systems in U.S. teaching hospitals and medical schools, and evaluates the utility of these systems in the U.S. healthcare system.

## 2. Materials and Methods

**Figure 1:**
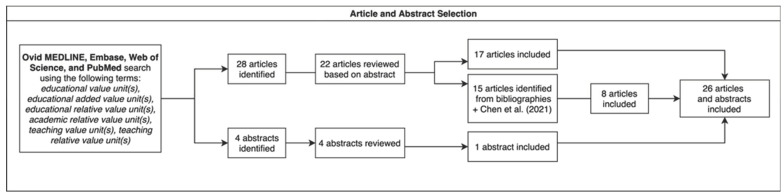
Method of article and abstract selection based on searches conducted in Ovid MEDLINE, EMBASE, Web of Science, and PubMed databases. Search results were examined to select articles and abstracts for review, and additional articles were identified from bibliographies of selected articles. Chen et al. (2021) [13] was selected for review independently of the above search due to prior knowledge of article content.

We conducted a narrative literature review to identify the inception of the EVU and to identify EVU systems implemented by U.S. academic medical centers, medical schools, and teaching hospitals. The searches were conducted using the Ovid MEDLINE, Embase, Web of Science, and PubMed databases. Search terms included “educational value unit(s)”; “educational relative value unit(s)”; “educational added value unit(s)”; “academic relative value unit(s)”; “teaching value unit(s)”; and “teaching relative value unit(s)”. The bibliographies of articles produced in this search were examined to identify additional studies and search terms. Studies meeting the following criteria were excluded: described EVU systems implemented in locations outside the U.S., described EVU systems in settings other than U.S. academic medical centers, and described relative value unit systems whose purpose was to reward any activities, including administrative, clinical, and educational tasks not currently receiving compensation under a work relative value unit system. We additionally excluded studies describing theoretical frameworks for EVU systems when studies describing the implementation and outcomes of EVU systems containing similar elements were available. There was no language restriction for the searches. In total, a combined 48 studies and abstracts pertaining to EVU systems were reviewed, and a combined 26 published studies and abstracts from 1999 to 2022 pertaining to EVU systems were included (Figure 1). Figures were created using the free, open-source software diagrams.net. 

## 3. Inception of the EVU

In 2000, the Association of American Medical Colleges (AAMC)’s Mission-based Management Program [6] first proposed the use of an Educational Relative Value Unit (RVU), also known as the EVU [10,11], Educational Added Value Unit (EAVU) [14], Educational Relative Value Unit (eRVU) [15], Teaching Value Unit (TVU) [16], Teaching Relative Value Unit (TRVU) [17], or Academic Relative Value Unit (aRVU or ARVU) [9,18], to provide medical institutions with a system by which they can measure faculty contributions to their educational mission. From our literature search, 21 U.S. teaching hospitals have published studies describing their EVU systems, which have come to serve several purposes. In specialties that do not offer reduced clinical hours or financial incentive for teaching activities, EVU systems can support physicians through financial compensation or through recognition of time spent on non-clinical activities [9]. These systems also offer an alternative to clinical productivity-based payment systems, which may deemphasize teaching in favor of increased clinical time. Furthermore, EVU systems serve to more evenly distribute teaching obligations and educational efforts among faculty members [11] and may be used to reallocate funds within [7] or between departments [8] to better support educational activities. Aside from altering compensation, EVU systems allow faculty members to track their educational activities, which is particularly useful for junior faculty members whose educational endeavors strongly influence their chance of promotion [9].

Though a variety of specialties may benefit from the use of an EVU system for the reasons mentioned above, self-described EVU systems have been implemented by Departments of Medicine [8], Emergency Medicine [9,10,11,16,17,19], Ophthalmology [13], Pediatric Surgery [18], Surgery [20,21], Pediatrics [22], Internal Medicine [7,23,24], Radiology [25], Anesthesiology [26], and Pathology [27], as well as Primary Care [28].

## 4. Calculating EVUs 

**Figure 2:**
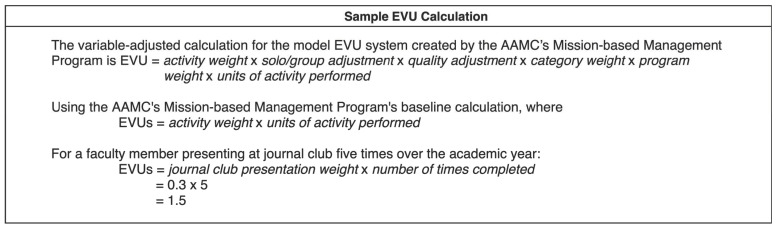
Sample EVU Calculation based on the EAVU Tracking Tool used at the Cincinnati Children’s Hospital Medical Center from Guiot et al. (2017) [14].

As described by the AAMC’s Mission-based Management Program, an EVU is defined as the value, or weight, assigned to different educational activities in a manner specific to a given institution. Educational activities which accrue EVUs place an emphasis on medical student and resident education and include, but are not limited to, developing a course, clerkship, or laboratory program, mentoring students, teaching, publishing peer-reviewed articles, serving in education administration, or achieving scholarship in education. The weight placed on these activities is institution specific and may be influenced by factors such as time and effort spent on an activity, the level of expertise required to perform an activity, and the degree to which the activity contributes to the school’s mission. The baseline calculation for the model EVU system created by the AAMC’s Mission-based Management Program is EVU = activity weight × units of activity performed. This equation can be modified with several variables, including a solo/group adjustment for the number of participating faculty, the quality of the activity, the category weight, or the program weight [6]. A sample calculation using the EVU system established by the Cincinnati Children’s Hospital Medical Center (CCHMC) is included below (Figure 2) [14].

Medical institutions vary in how they determine which activities accrue EVUs and what factors contribute to the EVU calculation. Most departments that have implemented EVU-based systems created special committees, comprised of individuals like educational faculty, physicians, division directors, and vice chairs, to compile and approve a list of educational and teaching activities that earn EVUs [7,8,9,10,11,13,14,15,16,22,27,29]. Committees may reference activities performed in the prior academic year [10] or use educational guidelines, like the American Academy of Medical Colleges Committee Educator Promotion categories [14], to create their initial list of activities.

While similar activities accrue EVUs across institutions, departments primarily differ in factors contributing to their EVU calculations. Many factor in preparation for a given activity [8,9,10,14,16,22,23,30], the effort displayed by faculty members [9,13,25], prestige garnered [16,20,27], learner type (medical student/resident versus fellow) [11], and the degree of seniority of faculty [20,29]. Other institutions may weigh their EVU activities depending on the relative importance or quality of the activity to learners and teachers, which can be discerned through administering surveys to medical students, residents, and faculty [27,29]. In these systems, learners may particularly benefit from the implementation of an EVU system because activities that are known to positively affect their future careers are assigned a greater weight. Departments may also add or subtract EVUs from a faculty member’s annual EVU count depending on their performance in a given activity [16] and do not assign specific weights to activities, instead focusing on time required to complete each activity [7,8]. Which factors and weights are incorporated into each institution’s EVU calculation is subjective and ultimately reflects its culture and educational priorities. See Table 1.

Institutions that have established EVU systems additionally differ in the EVU obligations assigned to their faculty. In many EVU systems, faculty members must spend a minimum number of hours performing educational activities. This minimum requirement may be influenced by how much annual teaching effort is available or how much teaching effort is necessary to achieve the department’s educational mission and may range from as little as 30 h [10] to 400 h [11]. Other systems simply assign a total number of EVUs to be accrued annually by each faculty member [15,16]. Often, the EVU obligation assigned to faculty members is impacted by the faculty member’s employment status, with full-time faculty members on educator career paths having larger EVU obligations than part-time faculty or fellows [11,15]. For mandatory EVU systems, failure to achieve one’s EVU obligations can result in funding cutbacks, increases in future teaching time, increases in clinical obligations, or reconsideration of one’s faculty status [11,16]. For voluntary EVU systems, failure to achieve one’s EVU obligations may result in a lack of or reduced financial bonus at the academic year’s end [9,13,19,22].

## 5. EVU Tracking Systems 

To keep track of annual EVU totals for a given academic year, departments may use a combination of self-reporting, record-keeping systems, and administrative data. Physicians may self-report educational activities to academic leadership who provide final approval or log their activities in systems like RedCap or Microsoft Excel [14,19]. Administrative data, such as conference schedules, attendance records, or Medhub reports [10], can also be used track educational activities. Some departments have also created web-based systems [20,25,29] or online dashboards to log educational activities and to calculate EVUs earned. Using one such system, faculty members received a monthly email detailing their EVU accruals [9].

## 6. EVU Systems and Financial Compensation 

Some U.S. teaching hospitals offer financial incentives for educational efforts performed under an EVU system [7,8,9,10,11,13,16,18,19,20,21,22,24,28,30]. Assuming a faculty member has met their baseline EVU requirements, this monetary benefit may be provided as a small percentage (≤5%) [10,11] of their annual or baseline salary, as a component of their annual financial bonus, or as a change in salary support [7,18,19,20,22,27]. Alternatively, faculty may be awarded a dollar amount per EVU [8,28,30], like at the Department of Medicine at the Medical University of South Carolina, where faculty were awarded USD 41.00 per EVU and 1 h spent on teaching = 2 EVU [8]. Departments may also compensate faculty based on their educational performance relative to their peers, where the financial bonus is either calculated using Individual EVUs earned/Group EVUs earned × teaching incentive dollars [16] or as lump sum amounts ranging from USD 0 to approximately USD 40,000 [13,19]. Due to the relatively small compensation earned for educational efforts under EVU systems compared to the potential bonuses unlocked by meeting clinical productivity criteria, it is unlikely that financial compensation plays a significant factor in the success of these systems. Rather, other drivers, namely advancement in faculty position, are likely responsible for the increased educational efforts made by faculty participating in these systems.

## 7. Outcomes of Implementing an EVU System 

Implementing an EVU system is an effective way to advance an institution’s educational mission because these systems successfully redistribute educational burden among faculty members, increase the number of faculty members who meet baseline educational and teaching expectations, and promote educational productivity. In the Department of Emergency Medicine at Johns Hopkins Medicine and in the Department of Internal Medicine at St. Joseph Mercy Hospital in Ann Arbor, teaching responsibilities were more equally distributed among faculty after the implementation of their EVU systems [11,23]. Similarly, the Departments of Emergency Medicine at the University of Michigan Medical School and the New York University School of Medicine saw a significant increase in the number of faculty who attended conferences (*p* < 0.005, *p* < 0.001) and in the number of completed resident evaluations (*p* < 0.005, *p* < 0.001) after incorporating EVU systems into their departments [9,10]. Educational productivity also increases under EVU systems, with some institutions reporting nearly a 30% increase in the mean individual productivity of faculty after implementation (*p* = 0.01) [16], others reporting a 10% increase (84% to 94%) in the number of faculty meeting baseline educational requirements [10] and still others reporting a statistically significant increase in annual peer-reviewed publications [18,20,26].

EVU systems additionally motivate physicians with greater clinical obligations to engage in teaching, mentorship, and leadership activities. Physicians with the highest clinical load in pediatric emergency medicine departments affiliated with Emory University had a significant increase in teaching productivity post-implementation of an EVU system (*p* = 0.03) [16], as did emergency medicine physicians at the University of Virginia School of Medicine (79% increase) [17]. Similarly, ophthalmologists at Weill Cornell Medicine engaged in significantly more mentorship (*p* = 0.013) after the implementation of their points system [13]. In the Department of Surgery at the Baylor College of Medicine, the number of faculty holding committee positions in academic organizations increased significantly after implementation of their EVU system (*p* < 0.001) [20].

EVU systems may additionally contribute to changes in external funding received by physicians and medical departments. One Department of Pediatric Surgery saw an increase in annual external federal funding from USD 750,168 to USD 5,768,243 during the period in which their EVU system was implemented [18]. In another surgical department, there was a statistically significant increase in total research funding, total NIH funding, total income from active grants, and income from industry sponsored trials after implementation of their EVU system (*p* < 0.001) [20]. This increase in extramural research funding also occurs in non-surgical specialties, such as emergency medicine. At Oregon Health & Science University, extramural research funding increased from USD 950,844 in the first year of their EVU system to USD 2,735,233 in the tenth year [19]. Though these departments saw an increase in external funding after implementation of their EVU systems, there are a variety of other factors that may contribute to this phenomenon, such as inflation or an increase in department size leading to a larger number of grant applications.

While EVU systems can lead to increases in external funding for research, they can also lead to shifts in salary within departments. In the Department of Internal Medicine at the University of Kansas School of Medicine, 60% of faculty had a mean salary support reduction of USD 28,814 after implementation of their system, while the remaining 40% had a mean salary support increase of USD 29,453. In total, the department shifted USD 1.66 million in funding among faculty members [7], with mixed responses from faculty: only 50% felt this shift in funds offset clinical salary losses incurred while engaging in educational activities [24]. In the Department of Medicine at the Medical University of South Carolina, over USD 500,000 was redistributed between departments over a four-year period based on the number of educational value units accumulated by each department. While physician salaries did not change under this system, the salary deficit between physicians in different departments decreased by more than USD 24,000, because physicians who engaged in educational efforts no longer received reduced salaries due to lower clinical productivity [8]. See Table 2.

## 8. Faculty Response to EVU Systems 

Though there is limited data assessing the reception of EVU systems, current literature suggests the faculty response to these systems is mixed. Some faculty feel quantifying academic efforts could negatively impact their careers if their degree of activity is seen as lacking, while others feel academic activities are specialty specific; and thus, generalized categories in EVU systems do not accurately reflect their level of engagement [29]. Faculty may additionally become upset when they do not meet baseline educational requirements, which in some cases, may lead to a departmental re-evaluation of the criteria used to determine EVUs [22]. Theoretical discussions of EVU systems have also proposed that these systems may introduce competition between members of a department rather than facilitate collaboration [31] due to the inherent nature of the system comparing one’s performance to that of one’s colleagues. In contrast, faculty at the University of Kansas Department of Medicine positively received their EVU system: the majority of faculty felt their system had a positive or neutral impact on their educational productivity, as well as on the quality and quantity of educational activities [24].

## 9. Limitations of an EVU System 

Some limitations exist with the implementation of EVU systems in U.S. teaching hospitals and medical schools. EVU systems do not always assess the quality of educational activities performed, so an increase in educational productivity may not reflect an equivalent increase in learning. However, the Department of Emergency Medicine at Johns Hopkins Medicine did not factor quality into their EVU calculations and still found equivalent learner satisfaction in educational programs before and after the implementation of their system [11]. If faculty in different departments do not equally participate in their institution’s EVU system, the system may falsely reflect educational effort made by departments as a whole, which could lead to an inappropriate redistribution of funds across departments. With some EVU tracking systems, there may also be self-report bias from physicians and faculty; and therefore, their EVUs may not be truly reflective of their educational effort. By setting baseline expectations, some EVU systems may also disincentivize some faculty from engaging in educational activities beyond their minimum expectations [15], though there is limited evidence of this occurring. 

## 10. Discussion 

Since its introduction in 2000 by the AAMC’s Mission-based Management Program, the EVU has been used across U.S. teaching hospitals and medical schools to assess faculty contribution to an institution’s educational mission. A limited number of studies describing current EVU systems have been published; however, the available literature supports the notion that these systems successfully advance an institution’s educational mission in a myriad of ways. EVU systems redistribute educational burden and funding among faculty, increase the number of faculty meeting baseline educational requirements, promote educational productivity, support research endeavors, and encourage physicians with greater clinical obligations to engage in teaching, mentorship, and leadership activities. Department chairs can use EVUs to evaluate departmental contributions to educational achievement relative to other departments and use this knowledge to adjust incentives for clinical, research, administrative, or teaching activities. Additionally, by recognizing and rewarding educational efforts, EVU systems may help promote faculty vitality and reduce burnout—particularly in the midst of the COVID-19 pandemic which is actively reshaping employee motivation and workforce behavior [32]. 

The implementation of EVU systems most greatly benefits academic physicians, junior faculty on an educational career path, and trainees at various stages. While EVU systems may encourage physicians with higher clinical loads to engage in more educational activity [16], they are unlikely to motivate those physicians who are already uninterested in contributing to a school’s academic mission. For faculty who dedicate a significant proportion of their careers to teaching and education; however, these systems provide recognition for their efforts and serve to reduce salary deficits incurred from lower clinical productivity. EVUs are of particular use to junior academic faculty, as they can be used to track the activities most likely to advance their career and may be used as a tangible, objective metric for promotion. Medical students, residents, and fellows also benefit from EVU systems since faculty are encouraged to engage in more teaching and research efforts, and many institutions have begun to factor learner satisfaction into their EVU weighting [27,29]. By incentivizing physicians to mentor and educate trainees, EVU systems facilitate the process by which clinical knowledge and physicianship is passed from one generation of physicians to the next. Though this study does not address EVU systems implemented in settings other than U.S. academic medical centers, such as psychology or dentistry, these systems may additionally benefit from an EVU system for the reasons mentioned above.

As mentioned previously, the monetary reward associated with EVUs is unlikely to be a significant factor contributing to the increased educational productivity seen under these systems. Faculty who already engaged in teaching, research, and administration will continue to perform these activities whether or not they accrue EVUs, especially under voluntary systems. Comparing a faculty member’s EVU totals to their peers can motivate some to give a greater educational effort; this motivation may be driven by a sense of responsibility, a desire to meet the educational mission of their institution, or feelings of shame.

## 11. Limitations 

This narrative review has several limitations. We limited our search to the Ovid MEDLINE, Embase, Web of Science, and PubMed databases to identify relevant studies and abstracts. Due to the search terms used, it is possible that we did not include the entire body of literature self-reporting EVU systems; however, we attempted to minimize this chance by examining the bibliographies of selected studies to identify additional search terms. We did not include studies discussing the implementation of EVU systems in non-medical settings, such as psychology or dentistry, or in locations other than the U.S. In addition, when discussing the outcomes of EVU systems, we are only able to imply association, not causation, as several factors may have played a role in the changes seen with the implementation of EVU systems. 

## 12. Conclusions

In conclusion, EVU systems are an effective way to advance an institution’s educational mission and reward individual and departmental educational efforts in U.S. academic medical centers and medical schools. As these institutions renew their commitment to their educational missions, EVU systems will likely become a permanent fixture in the evaluation and payment schemes of U.S. academic medical institutions.

## Figures and Tables

**Table 1:** Some Factors Contributing to EVU Calculations in Reporting U.S. Medical Institutions.

Factor	No.	Institutions
Preparation time	8	Department of Medicine, Medical University of South Carolina ^1^; Department of Emergency Medicine, University of Michigan Medical School ^2^; Ronald O. Perelman Department of Emergency Medicine, New York University School of Medicine; Department of Internal Medicine, St. Joseph Mercy Hospital; Departments of Pediatrics and Emergency Medicine ^3^; Cincinnati Children’s Hospital Medical Center ^4^; Department of Pediatrics, University of California Davis; Mount Sinai School of Medicine
Faculty effort	3	Ronald O. Perelman Department of Emergency Medicine, New York University School of Medicine; Department of Ophthalmology, Weill Cornell Medicine; Department of Diagnostic Radiology, University of Maryland School of Medicine
Prestige of activity	2	Department of Surgery, Baylor College of Medicine; Department of Pathology, Johns Hopkins Medicine
Learner type	1	Department of Emergency Medicine, Johns Hopkins Medicine
Degree of faculty seniority	2	Department of Surgery, Baylor College of Medicine; UC Davis Health System
Relative importance to learners	2	Department of Pathology, Johns Hopkins Medicine; UC Davis Health System
Activity time	2	Department of Internal Medicine, University of Kansas School of Medicine; Department of Emergency Medicine, University of Michigan Medical School ^2^

^1^ Only Grand rounds presentations were given EVU credit for preparation time. ^2^ EVU system implemented in both pediatric and adult divisions of Department of Emergency Medicine. ^3^ Departments of Pediatrics and Emergency Medicine affiliated with Hughes Spalding Children’s Hospital, Grady Health System, Emory University School of Medicine, and Children’s Healthcare of Atlanta. ^4^ System included faculty participants from hospital medicine, general and community pediatrics, emergency medicine, behavior medicine and clinical psychology, and biostatistics and epidemiology.

**Table 2:** Outcomes Associated with the Implementation of an EVU System in Reporting U.S. Medical Institutions.

Outcomes	No.	Institutions
Redistributed teaching responsibility	2	Department of Emergency Medicine, Johns Hopkins Medicine; Department of Internal Medicine, St. Joseph Mercy Hospital
Increased conference attendance	2	Department of Emergency Medicine, University of Michigan Medical School ^1^; Ronald O. Perelman Department of Emergency Medicine, New York University School of Medicine
Increased resident evaluation completion	2	Department of Emergency Medicine, University of Michigan Medical School ^1^; Ronald O. Perelman Department of Emergency Medicine, New York University School of Medicine
Increased educational productivity	3	Department of Medicine, Medical University of South Carolina; Department of Emergency Medicine, University of Michigan Medical School ^1^; Department of Pediatric Surgery, Nationwide Children’s Hospital
Increased number of annual peer-reviewed publications	3	Department of Pediatric Surgery, Nationwide Children’s Hospital; Department of Anesthesiology, University of Pittsburgh Medical Center; Department of Surgery, Baylor College of Medicine
Increased teaching productivity	2	Departments of Pediatrics and Emergency Medicine ^2^; Department of Emergency Medicine, University of Virginia School of Medicine
Increased mentorship	1	Department of Ophthalmology, Weill Cornell Medicine
Increased leadership in academic organizations	1	Department of Surgery, Baylor College of Medicine
Increased external funding	3	Department of Pediatric Surgery, Nationwide Children’s Hospital; Department of Surgery, Baylor College of Medicine; Department of Emergency Medicine, Oregon Health & Science University
Redistribution of funds within departments	1	Department of Internal Medicine, University of Kansas School of Medicine
Redistribution of funds between departments	1	Department of Medicine, Medical University of South Carolina

^1^ EVU system implemented in both pediatric and adult divisions of the Department of Emergency Medicine. ^2^ Departments of Pediatrics and Emergency Medicine affiliated with Hughes Spalding Children’s Hospital, Grady Health System, Emory University School of Medicine, and Children’s Healthcare of Atlanta.

## Data Availability

Not applicable.
